# Use of a MCL-1 inhibitor alone to de-bulk melanoma and in combination to kill melanoma initiating cells

**DOI:** 10.18632/oncotarget.8695

**Published:** 2016-04-12

**Authors:** Nabanita Mukherjee, Yan Lu, Adam Almeida, Karoline Lambert, Chung-Wai Shiau, Jung-Chen Su, Yuchun Luo, Mayumi Fujita, William A. Robinson, Steven E. Robinson, David A. Norris, Yiqun G. Shellman

**Affiliations:** ^1^ University of Colorado Anschutz Medical Campus, School of Medicine, Department of Dermatology, Aurora, CO, USA; ^2^ Institute of Biopharmaceutical Sciences, National Yang-Ming University, Taipei, Taiwan; ^3^ Division of Medical Oncology, University of Colorado Anschutz Medical Campus, School of Medicine, Aurora, CO, USA; ^4^ Department of Veterans Affairs Medical Center, Dermatology Section, Denver, CO, USA

**Keywords:** melanoma stem cells, cancer stem cells, drug-induced cell death, melanoma, SC-2001

## Abstract

MCL-1 (BCL-2 family anti-apoptotic protein) is responsible for melanoma's resistance to therapy. Cancer initiating cells also contribute to resistance and relapse from treatments. Here we examined the effects of the MCL-1 inhibitor SC-2001 in killing non melanoma-initiating-cells (bulk of melanoma), and melanoma-initiating-cells (MICs). By itself, SC-2001 significantly kills melanoma cells under monolayer conditions *in vitro* and in a conventional mouse xenograft model. However, even at high doses (10μM), SC-2001 does not effectively eliminate MICs. In contrast, the combination of SC-2001 with ABT-737 (a BCL-2/BCL-XL/BCL-W inhibitor) significantly decreases ALDH^+^ cells, disrupts primary spheres, and inhibits the self-renewability of MICs. These results were observed in multiple melanomas, including short term cultures of relapsed tumors from current treatments, independent of the mutation status of BRAF or NRAS. Using a low-cell-number mouse xenograft model, we examined the effects of these treatments on the tumor initiating ability of MIC-enriched cultures. The combination therapy reduces tumor formation significantly compared to either drug alone. Mechanistic studies using shRNA and the CRISPR-Cas9 technology demonstrated that the upregulation of pro-apoptotic proteins NOXA and BIM contribute to the combination-induced cell death. These results indicate that the MCL-1 inhibitor SC-2001 combined with ABT-737 is a promising treatment strategy for targeting melanoma.

## INTRODUCTION

For the first time several molecular-targeted and immunotherapy drugs are significantly improving the overall survival of patients with metastatic melanoma. However, despite these new therapeutics, many patients still do not improve or eventually relapse after the initial positive response [1, 2]. Thus, there is still a pressing need for continued research especially for the patients without the mutations targeted by these new drugs or those who relapse from these treatments.

MCL-1 (Myeloid cell leukemia sequence 1) is an anti-apoptotic protein of the BCL-2 family [3, 4]. The MCL-1 locus is one of the “top ten” most amplified genomic regions in a variety of human cancers [5], correlating with an upregulation of MCL-1 activity [6–10]. This increase in MCL-1 expression is often associated with chemotherapeutic resistance and relapse from therapeutics [3]. Pharmacological inhibition or the molecular down regulation of MCL-1 via RNA-interference has shown to promote apoptosis and/or overcome drug resistance in multiple cancers [11–16]. Therefore, MCL-1 is a high-priority therapeutic target for cancer treatment [4, 6, 17]. Oncogenic activation of BRAF signaling in melanoma upregulates MCL-1 expression and contributes to increased resistance to BRAF/MEK inhibitors and overall tumor progression and survival [18]. We and others have shown that the knockdown of MCL-1 sensitizes melanoma cells to various treatments, including BRAF or MEK inhibitors [19–23] and thus, targeting MCL-1 can be a new alternative for melanoma treatment.

Cancer Initiating Cells (CICs) are a subpopulation of cancer cells which have enhanced tumor initiation, progression, and chemo-resistance [24–26]. Recently, multiple groups provided evidence of Melanoma Initiating Cells (MICs), and suggest that MICs can contribute to resistance to treatment [27–30]. Ideal cancer treatment strategies stress on eliminating the resistant subpopulations, such as CICs as well as the bulk of the tumors (non-CICs) cells to prevent relapse [29, 31].

In this study we tested the efficacy of an MCL-1 inhibitor, SC-2001, either alone or in combination with ABT-737 in killing melanoma cells and MICs. SC-2001 is a novel MCL-1 inhibitor, and it is structurally related to obatoclax, a small molecule inhibitor for multiple anti-apoptotic BCL-2 family members, including MCL-1. SC-2001 has anti-tumor activity for liver cancers and breast cancers [32–34]. ABT-737 is a small molecule BCL-2/BCL-XL/BCL-W inhibitor and has shown a promising result in cancer therapy either by itself or in combination with other chemotherapeutics in pre-clinical stage [35, 36]. However, many labs including ours have found that ABT-737 by itself is not very effective for treating melanoma as a single agent [23, 37, 38]. The results here suggest that the use of a combination of MCL-1 and BCL-2 inhibitors to induce NOXA and BIM is a promising treatment strategy for melanoma regardless of the mutation status of BRAF or NRAS, and it may overcome melanoma's resistance to current treatments.

## RESULTS

### MCL-1 protein expression is higher in melanomas compared with Melanocytes

**Figure 1:**
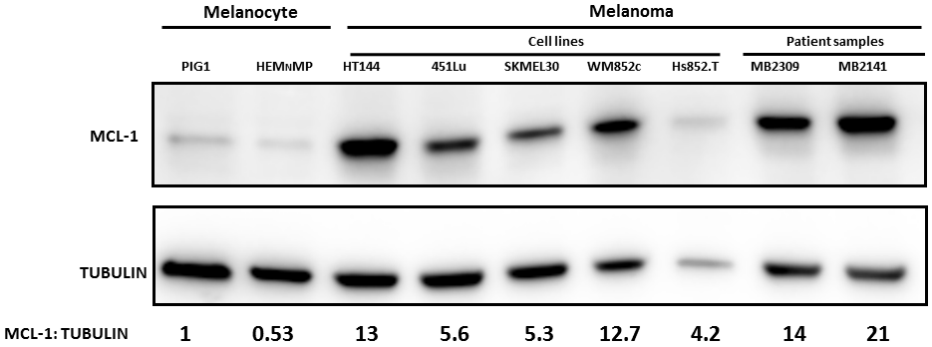
Higher MCL-1 protein expression in melanomas compared with Melanocytes Immunoblot of MCL-1 expression in lysates from melanoma cells and melanocytes cultured in sphere condition. Melanomas include common cell lines and short term cultures of melanoma samples relapsed from current treatments, with BRAF-mutated (HT144, 451Lu, and MB2309), NRAS-mutated (SKMEL-30, WM852c and Hs852T), NF-null (Hs852T), or wild-type for common mutations in BRAF, NRAS, or NF1 (MB2141).

MCL-1 expression is associated with chemotherapeutic resistance and relapse in various cancers [3] and its increased expression is correlated with melanoma progression [39]. However, it has not been examined whether this correlation remains consistent for the common mutations associated with melanoma. Here, the melanomas we tested include commonly used melanoma cell lines and tumor samples from melanoma patients. All tumor samples were maintained in either short term cultures *in vitro* or exclusively in a patient-derived xenograft model (PDX) (Figure 1). The melanomas used here include BRAF-mutant cells (HT144, 451Lu and MB2309), NRAS-mutated cells (WM852c, SKMEL-30, Hs852T), NF-null cells (Hs852T), or wild-type cells for the common mutations in BRAF, NRAS, or NF1 (MB2141). The last category has been referred to as triple-WT [40]. Most of the patient tumor samples used here have relapsed from the molecular-targeted treatment (MB2309) or treatments of multiple chemotherapies and radiation (MB2141). Figure 1 shows higher MCL-1 protein expression in multiple melanoma sphere cultures compared to normal human primary melanocytes (PIG1, HEM_N_MP), regardless of mutation status of these melanoma cultures. The increase in MCL-1 expression was between 4 to 21 fold. This finding provides the rationale to treat a broader range of melanomas with MCL-1 inhibitors to try to overcome resistance to current treatments. Therefore, we tested the efficacy of the MCL-1 inhibitor, SC-2001.

### SC-2001 is capable of eliminating the bulk of melanoma cells *in vitro* and *in vivo*

**Figure 2:**
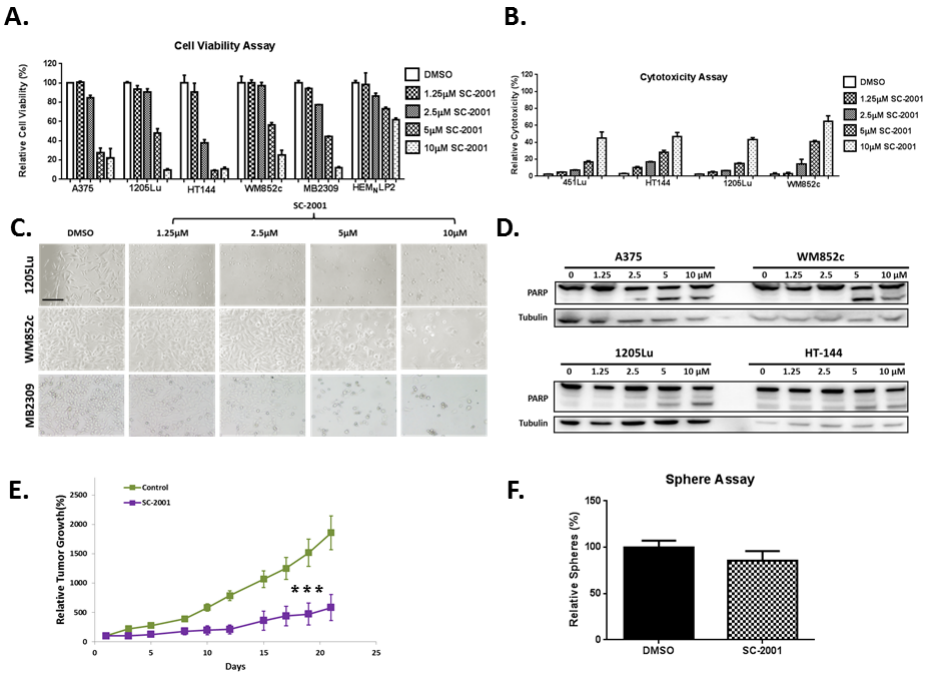
SC-2001 is capable of targeting the bulk of melanoma cells *in vitro* and *in vivo*, however it did not target the MIC population **A**. ATP assays with BRAF-mutated (A375, 1205-Lu, HT144 and MB2309), NRAS-mutated (WM852c) melanomas and melanocyte. At doses of 2.5uM or higher, SC-2001 reduced cell viability for all melanoma cell lines significantly compared to control (P<0.01 or less). **B**. Cyto-tox Glo assays with indicated melanomas. The drug, at doses of 5uM or higher, also showed increased cytotoxicity in a dose-dependent manner for all four melanoma cell lines tested compared to control (P<0.001 or lesser), regardless of their mutation status. **C**. Bright-field images of melanoma cells treated with increasing concentrations of SC-2001. Scale bar = 100μm. A bigger version of Figure 2C is provided in the Supplementary Figure S1. **D**. Immunoblot of full length and cleaved PARP for cell lysates treated with indicated doses of SC-2001. For (A) to (D), the cells were treated with the indicated concentration of SC-2001 for 48 hrs before being subjected to respective assays. **E**. Relative tumor volumes in a mouse xenograft model. The tumor volume at day 0 was set as 100%. SC-2001 significantly decreased the rate of tumor growth compared to the control (P <0.001). **F**. Sphere assays with the tumor cells collected at the end of the xenograft experiment from panel E. The tumor cells were not treated with any drugs during the sphere assay. No significant difference in number of spheres formed between the SC-2001 and DMSO treated samples. ***indicates P<0.001 or less; **indicates P<0.01.

To investigate whether SC-2001 affects cell viability, we used an ATP assay. SC-2001 significantly reduced cell viability in a dose-dependent manner in multiple melanoma cultures and only a minimal reduction for melanocytes (HEM_N_LP). At doses of 2.5uM or higher, SC-2001 reduced cell viability for all melanoma cell lines significantly compared to DMSO (P<0.01 or less). The melanoma cells included BRAF or NRAS mutated cell lines and tumor samples from melanoma patients that have relapsed from current treatments (Figure 2A). The IC50s for SC-2001 are 2.14μM to 4.62μM for the melanoma cell lines (Supplementary Table S1). The drug also showed increased cytotoxicity (at dose of 5 or 10 μM) in a dose-dependent manner for all four melanoma cell lines tested (P<0.001 or lesser) compared to DMSO, regardless of their mutation status (Figure 2B). Higher doses of SC-2001 (5 or 10 μM) resulted in a more rounded morphology or complete detachment from the plates (Figure 2C and Supplementary Figure S1) that is consistent with the ATP and cytotoxicity data suggesting that SC-2001 induced killing in these melanoma populations. Additionally, cleavage of PARP (Poly ADP-ribose polymerase 1) is a well-known marker of cells undergoing apoptosis [41], and we performed immunoblot assays of PARP to further assess the effects. Figure 2D shows that the higher doses of SC-2001 (5 or 10 μM) induced an increased level of cleaved PARP in all the melanoma cell lines tested. Additionally, an Annexin V assay demonstrated that SC-2001 (2.5 or 5 μM) caused dramatic apoptosis, ranging from ~35-80%, in all eight melanoma cell lines/patient samples tested relative to the DMSO control (P < 0.01 or less, Supplementary Figure S2). However, the SC-2001 treatment had only a modest effect on melanocytes (HEM_N_MP) (Supplementary Figure S2). Furthermore, in a conventional mouse xenograft model, SC-2001 significantly decreased the rate of tumor growth compared to the control (P <0.001) (Figure 2E).

### SC-2001 does not target the MIC population

Multiple studies have identified subpopulations of melanoma cells that possess characteristics of CICs including increased resistance to treatments [28, 30, 37, 42]]. One of the best *in vitro* methods used to study CICs is the sphere formation assay [6] and it has been successfully used to study MICs in many studies [37, 43–46]. Melanoma-spheres display stem cell like functions including self-renewability and tumorigenicity [46]; thus they can be used as a tool to enrich the cancer cell population that exhibits stem-like features for testing the potency of cancer drugs [25, 26, 47]. The primary sphere assay helps enrich the MIC population while a secondary sphere formation assay is an assay for measuring the self-renewal capacity of MICs *in vitro* [37]. To determine whether SC-2001 treatment eliminated both the bulk tumor cells and MIC population in the experiment described in Figure 2E, we performed sphere-forming assays starting from single cell suspensions isolated from the aforementioned experiment. We found that there was no significant difference in number of spheres formed between the SC-2001 and DMSO treated samples indicating that although SC-2001 could shrink the tumor in a conventional xenograft model, it was unable to effectively eliminate all the MICs (Figure 2F).

### Low dose SC-2001 plus ABT-737 targeted the MIC population regardless of their mutation status

**Figure 3:**
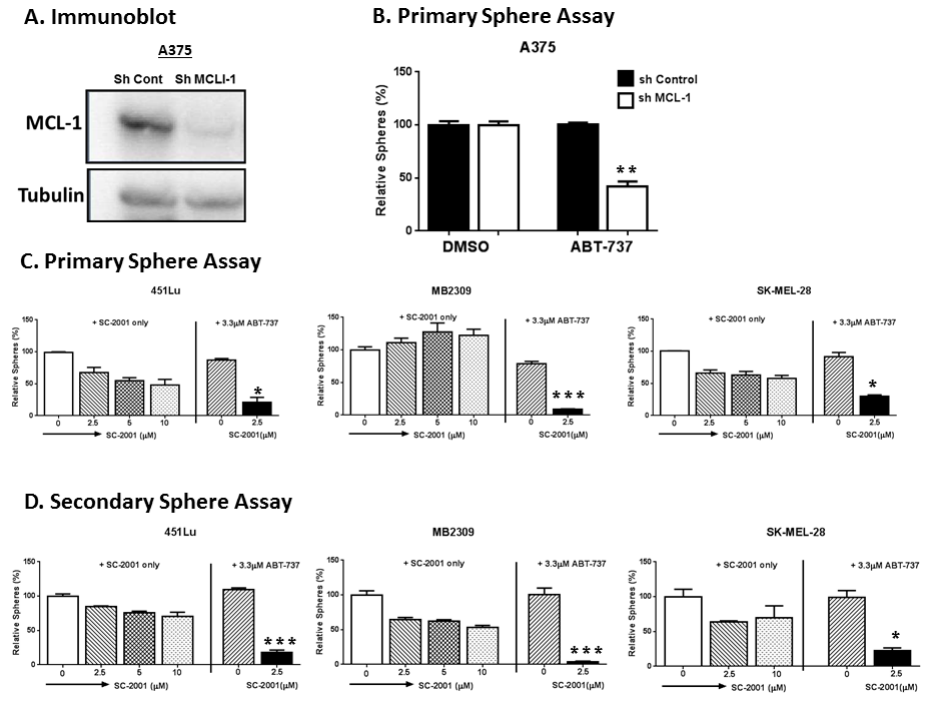
SC-2001 did not target the MIC population even at a high concentration (10 μM), while a lower concentration of SC-2001 (2.5 μM) combined with ABT-737 targeted the MIC population **A**. and **B**. A375 cells, stably carrying either control shRNA (sh-Control) or shRNA against MCL-1 (sh-MCL-1), were subjected to: (A) Immunoblot for MCL-1 to confirm the knockdown; or (B) Primary sphere assays. There was significant decrease in the number of spheres in sh-MCL-1 group, when treated with ABT-737 (P<0.01). **C**. Primary sphere assays. For primary sphere assay in (B) and (C), the spheres were treated with the indicated concentration of drug for 48 hrs before counting. **D**. Secondary sphere assays to test the efficacy of SC-2001 in targeting MIC by itself or in combination with ABT-737 with indicated melanoma cells. The combination inhibited the formation of both the primary (C) and secondary spheres (D) compared to DMSO, ABT-737 or SC-2001 only (2.5, 5 or 10 μM) (P < 0.05 or less). ***indicates P<0.001 or less; **indicates P<0.01; *indicates P<0.05.

Therapeutics with single molecular targets often fail in cancer therapy, and the CIC populations are thought to be reason for this. Thus, utilizing combination therapies that eliminate this resistant cell population is an emerging strategy to treat cancer [24, 48]. Many studies, including ours, have shown that targeting single anti-apoptotic BCL-2 family members is not sufficient to treat melanoma [23, 37, 38], and that targeting both MCL-1 and BCL-2 is needed to eliminate the MIC population [37]. We assessed if decreasing MCL-1 expression by shRNA can synergize with a BCL-2 inhibitor to abolish the MIC population in a sphere formation assay (Figure 3A and 3B). Knockdown of MCL-1 (shMCL-1) by itself, did not cause a significant decrease in the number of spheres compared to the shControl. However, when shMCL-1 cells were treated with the BCL-2 inhibitor ABT-737, there was a significant decrease in the number of spheres (P<0.01) (Figure 3B). This suggested that SC-2001 when combined with ABT-737 can be an effective strategy to target the MICs.

We therefore examined the efficacy of SC-2001 by itself and in combination with ABT-737 against MICs using sphere forming assays on multiple melanoma cell lines (Figure 3C and 3D). SC-2001 by itself did not significantly inhibit either primary or secondary sphere formation even at high concentrations (5 or 10 μM) compared to DMSO. On the other hand, when combined with ABT-737 (3.3 μM), it was very effective even at a lower concentration (2.5 μM). The combination inhibited the formation of both the primary and secondary spheres compared to DMSO or SC-2001 only (2.5, 5 or 10 μM) (P < 0.05 or less) (Figure 3C and 3D). This decrease is comparable to the effect observed for ABT-737 treatment on sh-MCL-1 cell lines.

**Figure 4:**
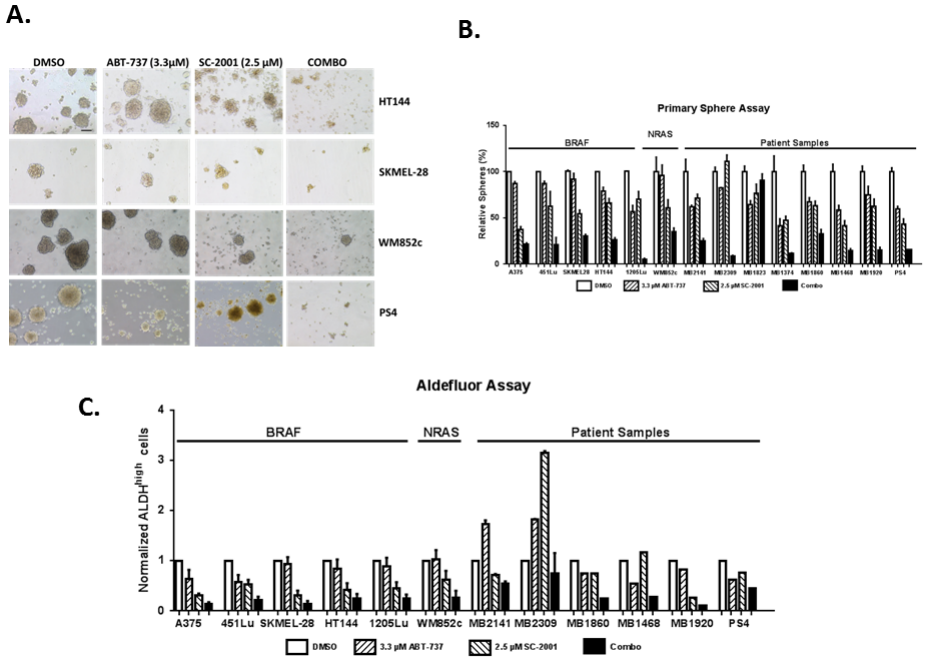
SC-2001 combined with ABT-737 targeted the MIC population of melanoma cells regardless of the mutation status Melanoma spheres were treated with indicated compounds, and then subjected to **A**. Bright field analysis, Scale bar = 100μm; **B**. Quantification of the number of primary spheres; For primary sphere assay, the spheres were treated with the indicated concentration of drug for 48 hrs before counting. The combination severely disrupted the primary spheres compared with the control, ABT-737 or SC-2001 treatment alone (P< 0.05 or less) regardless their mutation status in thirteen out of fourteen lines. The p-values for all comparisons are separately listed in Supplementary Table S2. **C**. Quantification of ALDH assay. For ALDH assay, the cells were treated with the indicated concentration of drug for 48 hrs before conducting the assay. The combination of SC-2001 and ABT-737 significantly decreased the percentage of ALDH^high^ cells compared with the DMSO control and ABT-737 alone regardless their mutation status (P < 0.05 or less) in seven lines. Unfortunately, we did not have enough material to do additional replicates for last four samples, so we could not statistically analyze the data. The p-values for all comparisons are separately listed in Supplementary Table S3.

To further examine the effects of this combination, we extended these assays to more melanoma samples, particularly the short term cultures of tumor samples from melanoma patients (Figure 4). Again, the samples tested here include BRAF or NRAS mutated, as well as wild type for BRAF, NRAS and NF1 (triple-WT) lines. The patient samples include the ones relapsed from current treatments. The spheres were allowed to grow up to reasonable size until Day 5 after seeding before being treated with single or combination drugs for 48 hrs. In thirteen out of fourteen samples, the combination severely disrupted the primary spheres compared with the control, ABT-737 or SC-2001 treatment alone (P< 0.05 or less, Figure 4A and 4B) regardless their mutation status. The p-values for all comparisons are separately listed in Supplementary Table S2. Additionally, we performed immunoblot of PARP cleavage, and further confirmed that the combination treatment induced apoptosis in all the melanoma samples tested (Supplementary Figure S3).

Multiple groups have found a positive correlation between cancer cells expressing higher aldehyde dehydrogenase (ALDH) and tumor formation efficiency including melanoma [43, 45]. We and others have previously established that cells with higher ALDH activity are enriched with MICs [37, 43, 45]. Therefore, we used an Aldefluor assay as an additional tool to examine the effects of the combination treatment on the MIC population (Figure 4C). In seven out of eight melanoma cell lines with enough cells for statistical analyses, the combination of SC-2001 and ABT-737 significantly decreased the percentage of ALDH^high^ cells compared with both the DMSO control and ABT-737 alone regardless their mutation status. (P < 0.05 or less) (Figure 4C). The p-values for all comparisons are separately listed in Supplementary Table S3. Interestingly, some of the patient samples showed an increased percentage of ALDH^high^ cells compared with the DMSO control in response to the single drug treatment. Unfortunately, we did not have enough material for the last four patient samples to perform enough assay replicates for statistical analyses. However, the overall trend was similar relative to the other cell lines.

Taken together, the sphere assay and the ALDH assay demonstrate that the combination of MCL-1 with BCL-2 inhibitors is better than either single drug to kill the MICs.

### SC-2001 combined with ABT-737 inhibited the self-renewability of MICs

While the primary sphere-forming assay enriches the population for stem-like cells, the secondary sphere assay measures the cell population's ability to regenerate after drug treatment [46]. This was done by conducting the primary sphere assay as described earlier, dissociating the primary spheres into single cells, and then replating the cells at the same viable cell density. However, we did not add additional drugs during the secondary sphere portion of the assay. This assay specifically assesses if any of the remaining viable cells—those that escaped chemotherapeutics—are capable of self-renewing and regenerating into a mass of tumor cells.

**Figure 5:**
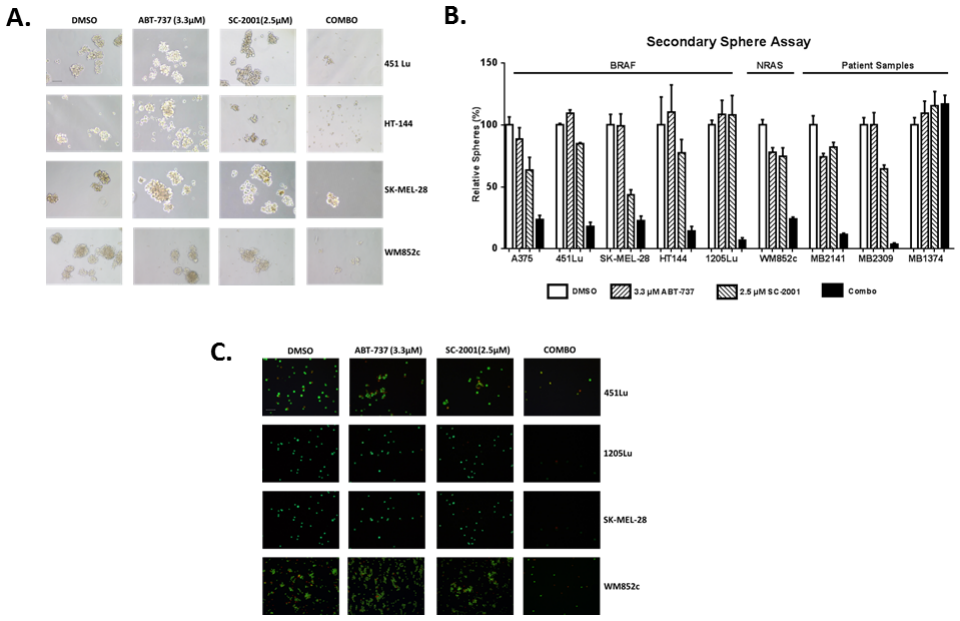
A low concentration of SC-2001 (2.5 μM) combined with ABT-737 inhibited the self-renewability of MICs Secondary sphere assays with the melanoma cells **A**. Bright-field images of secondary spheres. Scale bar = 100μm **B**. Quantification of the number of secondary spheres. The combination treatment significantly decreased the number of secondary spheres formed compared with DMSO, ABT-737 or SC-2001 treatment alone (P < 0.05 or less) in eight out of nine lines tested. The p-values for all comparisons are separately listed in Supplementary Table S4. **C**. Visualization of the secondary sphere cells with the Ethidium Bromide/Acridine Orange (EtBr/AO). Scale bar = 100μm. The cells were not treated with any drugs during the secondary sphere assay.

The combination treatment almost eliminated all secondary sphere formation in eight out of nine melanoma cell lines tested (Figure 5). Statistical analyses indicated that the combination treatment significantly decreased the number of secondary spheres formed compared with DMSO, ABT-737 or SC-2001 treatment alone (P < 0.05 or less) in eight out of nine lines tested (Figure 5A and 5B). The p-values for all comparisons are separately listed in Supplementary Table S4.

Visualization of these cells with EtBr/AO staining indicated that the majority of the cells in the control or single drug treatments were alive, but the majority of cells in the combination treatments were dead (Figure 5C). Thus, these results showed that the combination prevented the formation of secondary spheres and therefore decreased MIC's self-renewal capability in multiple cell lines.

### The combination of SC-2001 and ABT-737 inhibits the MIC-mediated tumor formation *in vivo*

Conventional mouse xenograft studies implant tumor cells at a very high cell number (> 1 million tumor cells) and cannot reasonably assess the efficacy of a drug in preventing the tumor initiating ability of CICs. Recently, Hirata and colleagues examined the effects of the transcription factor SphK1 on tumor initiating ability of breast CICs by injecting a small amount (100,000 viable cells) of a CIC-enriched cell population into a mouse xenograft model [49]. Similar approaches have been used previously in assessing tumor-initiating ability in other cancer stem cell studies [49–52]. Here, we employed a similar xenograft method with a low cell number implantation to test the differences in tumor-initiating-ability between the control and treatment groups. This xenograft method makes testing cancer initiation more feasible than the standard series dilution method. We also used melanoma cells derived from patients whose tumors eventually relapsed from both BRAF/MEK inhibitors and immunotherapy treatment (MB1860). These tumors were only maintained and passaged in the PDX model prior to this experiment.

**Figure 6:**
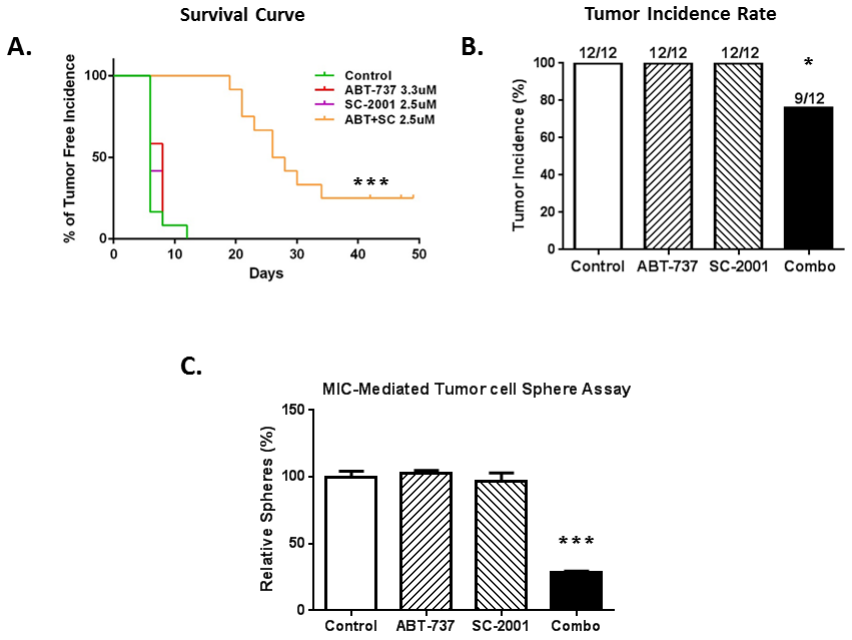
The combination of SC-2001 and ABT-737 inhibited the MIC-mediated tumor formation *in vivo* The treatment effects were tested in a xenograft model initiated with a low-number of MIC-enriched cells. For tumor injection, we used the surviving cells from the sphere cultures, upon treatments of SC-2001 and ABT-737, either alone or in combination, at the density of 50,000 viable cells per injection. **A**. Tumor-free survival curve shows a significantly longer tumor–free time in the combination group, compared to the vehicle or single drug group (P<0. 001). **B**. The percentage of tumor incidence was significantly lower in the combination group compared to control or single drug groups (P<0.05). **C**. Sphere assays with the tumor cells collected at the end of the animal experiment, and the number of spheres was significantly lower in the combination group compared to control or single drug groups (P< 0.001). N=3. ***indicates P<0.001 or less; *indicates P<0.05.

We first treated sphere culture samples from a relapsed patient with DMSO, single, or combined drugs for 48 hrs *in vitro*. After the treatment, we implanted 50,000 surviving viable cells from each group into mice and then monitored the tumor growth *in vivo* as a readout for the impact of each treatment on tumor-initiation ability. Figure 6A shows that the combination group mice had the longest tumor-free survival time compared to vehicle as well as single drug group (P<0.001). Tumor incidence rate was calculated as the number of tumors generated /number of implantations expressed as percentage. Tumor incidence rate was significantly lower in the combination group compared with the vehicle or single treatment group (P < 0.05) (Figure 6B). These results suggest that the combination-treated populations contained fewer MIC-like cells. To further confirm this, we performed sphere-forming assays with the single cell suspensions isolated from the surviving tumors, and found that the combination significantly reduced the number of spheres compared with vehicle or individual treatments (P < 0.001) (Figure 6C). There was no significant difference between the control and single-drug treated groups in all the above three analyses. Taken together, results here suggest that the combination reduced the MIC population.

### The SC-2001 and ABT-737 combination induces NOXA- and BIM-mediated killing of MICs

**Figure 7:**
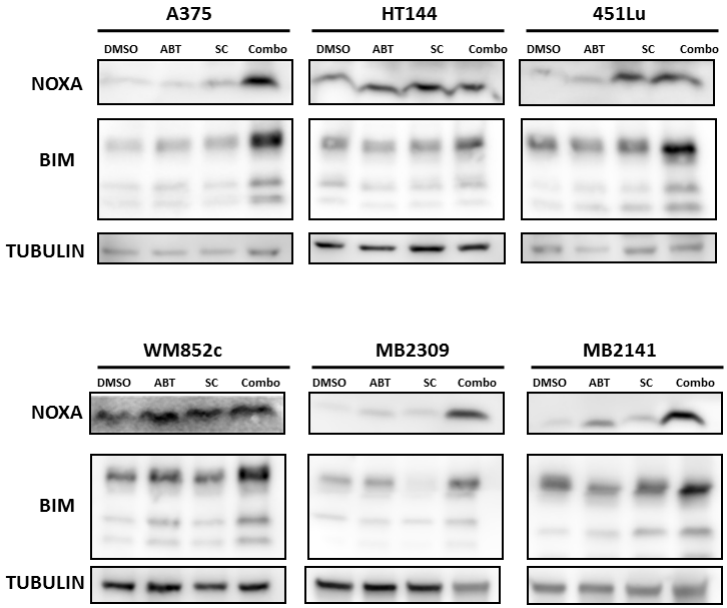
Combination induced NOXA- and BIM-mediated killing of MICs Immunoblot of the lysates harvested from cells treated with indicated drugs for 48 hrs in sphere conditions.

We have found previously that BH3 only pro-apoptotic proteins, NOXA and BIM, played a crucial role in inducing cell death when ABT-737 was used in combination with other agents [23, 37, 38]. These two proteins are also known to play important roles in regulating MCL-1 and BCL-2 in melanoma [37]. Thus, to evaluate the mechanisms behind the combination-induced cell death, we performed immunoblot studies to examine the expression of these two proteins. We found that the combination treatment notably increased the expression of both NOXA and BIM (Figure 7). Results were similar in BRAF mutated (A375, HT144 and 451Lu), NRAS mutated (WM852c), and patient melanoma cells (MB2309 and MB2141) (Figure 7).

**Figure 8:**
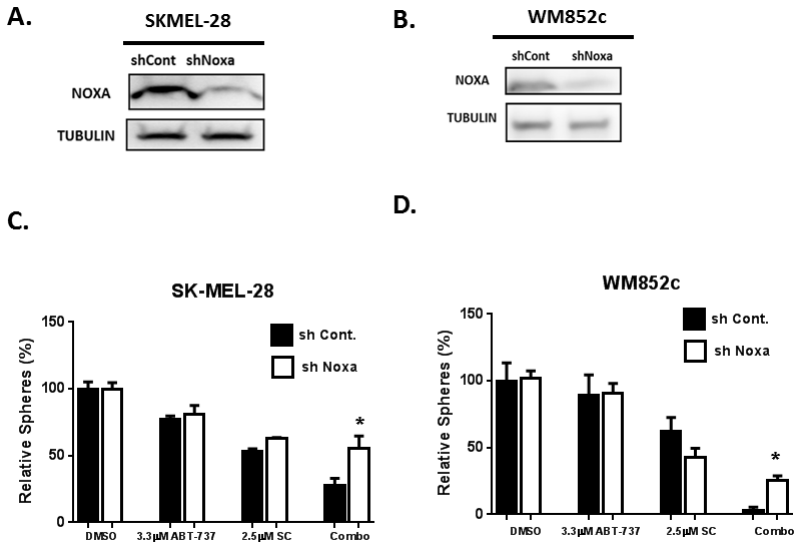
Knock-Down of NOXA protected against combination-induced cell death Immunoblot to confirm the NOXA knockdown for SKMEL-28 **A**. and WM852c **B**. Sphere assays were performed with melanoma cells stably carrying either control shRNA (sh control) or shRNA against NOXA (shNOXA): **C**. SK-MEL-28 and **D**. WM852c. For primary sphere assay, the spheres were treated with the indicated concentration of drug for 48 hrs before counting. Knockdown of NOXA significantly protected the cells against the combination-induced disruption of spheres (P<0.05). *indicates P<0.05.

To further determine whether this induction of NOXA or BIM contributes to killing effects of the combination, we examined the effects of knocking-down NOXA or knocking-out BIM on the killing potency of the combination of SC-2001 and ABT-737 on cells. We have established stable cell lines with NOXA knockdown using an shRNA-mediated approach [23], and found that knockdown of NOXA significantly protected against the combination-induced disruption of spheres (P<0.05) (Figure 8A to 8D) in both BRAF and NRAS mutated melanoma cell lines.

**Figure 9:**
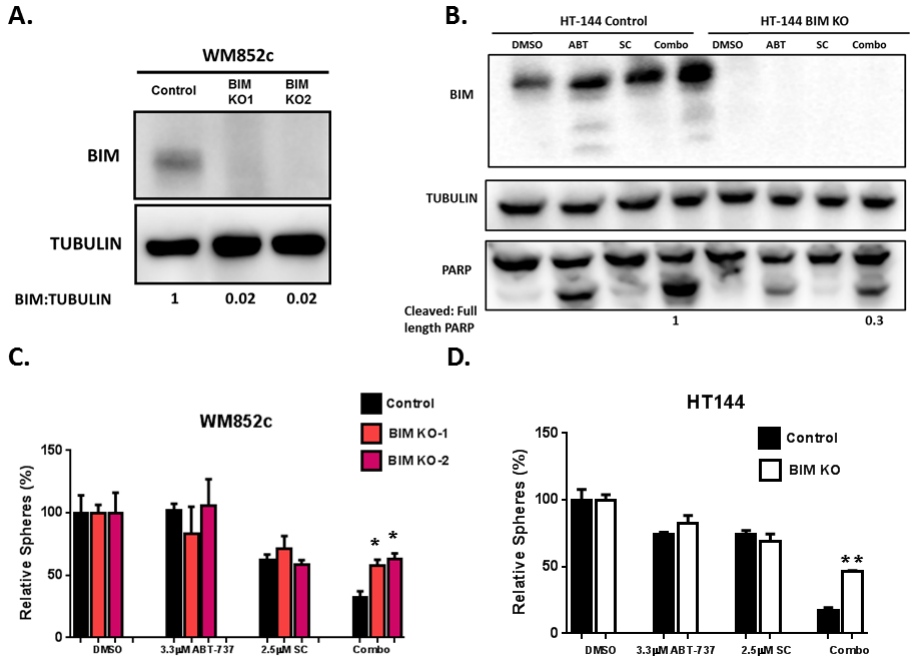
Knock-out of BIM protected melanoma cells against combination-induced cell death WM852c and HT144 melanoma cell lines with BIM knockout were generated with the CRISPR/Cas9 system. Knockout of BIM were determined by Immunoblots **A**. and **B**. and these cells were used for sphere assays **C**. and **D**. (B) also shows the full length and cleaved PARP from the HT144 cells, with either control or CRISPR-mediated knockout of BIM, upon indicated treatments in sphere conditions. The ratio of Cleaved/full length PARP of the cells treated with combination was quantified, and the one in the control cell was set as 1. (C) and (D) show the sphere assays with the knockout clones (KO1 and KO2) of WM852c cells with indicated drug treatments for 48 hrs. KO of BIM significantly protected the cells against the combination-induced disruption of spheres (P<0.05 or less) in both WM852c and HT144. **indicates P<0.01; *indicates P<0.05.

In previous studies, we found the effects of knocking-down BIM varied depending on the cell lines used, probably due to incomplete knock-down of BIM [37]. This is one of the limitations for shRNA technology since the levels of knock down are achieved with variable efficiency in most cases. Recently, the CRISPR/Cas9 system has been successfully developed for genome engineering/editing and is one of the most innovative and revolutionary methods currently available for modifying human genome [53, 54]. This enables making modifications at the DNA level with permanent and heritable changes in the genome much more feasible [55]. Thus we decided to knockout BIM expression using the CRISPR-Cas9 technology (Figure 9). We used the same system/vectors from Dr. Zhang's group [56], which have been used in a human melanoma cell line to screen for genes that are involved in resistance to BRAF inhibitor treatment [53, 54].

We successfully established multiple clones with complete reduction of BIM protein expression in melanoma cell lines with the CRISPR system (BIM KO) (Figure 9A and 9B). Sphere-forming assays demonstrated that KO of BIM also significantly protected the cells against the combination-induced disruption of spheres (P<0.05 or less) in both BRAF and NRAS mutated melanoma cell lines (Figure 9C and 9D). In addition, Immunoblot assays also indicated that the knockout (KO) of BIM reduced the drug combination-induced PARP cleavage and dramatically decreased the ratio of cleaved/full-length PARP (Figure 9B). These data suggest the combination-induced killing is both NOXA- and BIM- dependent.

## DISCUSSION

This study is the first to examine the efficacy of MCL-1 and BCL-2 inhibitors in combination to induce killing of both bulk melanoma cells and MICs. Consistent with other reports we found higher endogenous MCL-1 expression in most melanomas compared to melanocytes in both cell lines and tumor samples from patients (Figure 1) [21, 39]. This data also suggests that the higher expression of MCL-1 in melanomas is independent of the mutation status for BRAF or NRAS. Therefore, the anti-apoptotic protein MCL-1 is a potential treatment target for a broad range of melanomas.

We first tested the effects of the novel MCL-1 inhibitor SC-2001 in multiple assays *in vitro* and in a conventional xenograft mouse model *in vivo*. SC-2001 by itself eradicated the bulk of melanoma cells *in vitro* regardless of the mutation status and inhibited tumor growth *in vivo* (Figure 2). In this study, we use the term “de-bulk” to describe the targeting of non-cancer-initiating cells, as in many other manuscripts in the research field of cancer stem/initiating cells [57–60]. Taken together, these results indicate that the SC-2001 could be a promising treatment option to de-bulk melanomas *in vitro* and *in vivo*.

Despite the efficacy of SC-2001, it was unable to target the MIC population (Figure 2F). This suggests that combinatorial treatments may be required to eliminate the resistant subpopulations of melanoma such as MICs. In support of this idea, we have demonstrated here that knocking down MCL-1 sensitizes the MIC population to ABT-737 (Figure 3A and 3B). This supports the hypothesis that combining MCL-1 and BCL-2 inhibitors will effectively target the MIC sub-population.

*In vitro*, we tested this hypothesis utilizing primary/secondary sphere formation assays and an ALDH activity assay to assess the effects of this combination on MICs from multiple melanoma cultures (Figure 4 and 5). It is well established in the cancer-stem-cell field that the primary sphere assay and ALDH assay measure the relative amount of MIC cells while the secondary sphere assay measures the capacity of self-renewal ability [43, 45, 46, 61]. The combination of SC-2001 and ABT-737 effectively eliminated the MIC population regardless of the mutation status in the primary sphere assay (Figure 4). Additionally, only the combination treatment significantly decreased self-renewal capacity by inhibiting the re-growth of tumor cells in the secondary sphere assay (Figure 5). Excitingly, these results were observed even in the melanomas that have relapsed from molecular-targeted or immune-therapies and also those with varying genetic backgrounds (wild type or mutated for BRAF, NRAS, or NF1). This data collectively suggests that this combination effectively targets the MIC population and may overcome melanoma's resistance to current therapies.

We further explored the effects of the SC-2001 and ABT-737 combination on the tumor initiating ability of the MIC population *in vivo* using a modified xenograft model. This was initiated with a small amount of remaining viable cells from MIC-enriched populations after being treated to test for differences in tumor initiating ability. We found that only the combination treatment significantly increased the duration of tumor-free time and decreased tumor incidence (Figure 6A and 6B). This result was further confirmed using secondary sphere-forming assays with single cell suspensions isolated from the surviving tumors (Figure 6C). These results suggest that the combination significantly decreased the MIC-mediated tumor initiating ability *in vivo*. This further supports that an MCL-1/BCL-2 co-targeting strategy could be beneficial for melanoma patients, especially those relapsed from other treatments.

Despite the high efficacy of this combination in the experiments reported here, not all patient samples that were tested responded to the combination treatment. Sample MB1823 was resistant to the combination in the primary sphere assay, and MB1374 was resistant in the secondary sphere assay. Further studies are needed to determine why those two tumor samples did not respond and to identify any potential biomarkers that could be predictive of the success of this combination treatment. Moreover, CICs are not the only factors contributing to tumor heterogeneity or resistance to treatment, and others may include cellular plasticity and phenotypic switching [62–65]. Future studies are needed to examine how this combination treatment may affect these aspects of melanoma.

In addition to inhibiting MCL-1, SC-2001 has been reported to induce cell death through SHP-1 dependent STAT3 inactivation in liver and breast cancer cells [33, 34]. However, we did not detect any consistent changes in SHP-1 or STAT3 upon treatment in our experiments (data not shown). Instead, one of the main mechanisms responsible for the combination induced MIC death observed here is the up-regulation of the pro-apoptotic proteins NOXA and BIM (Figure 7). In support of this idea, an increase in the expression of NOXA and BIM occurred upon treatment in both mutated BRAF and NRAS cell lines along with melanoma patient samples (Figure 7). In addition, the knock-down of NOXA (shRNA) or the knock-out of BIM (CRISPR-Cas 9 technology) significantly protected against combination-induced cell death (Figure 8 and 9). The protection we have seen for loss of either NOXA or BIM alone was 23-28%, which was commonly seen in other studies with BCL-2 family members [37, 66–68]. Multiple members in the BCL-2 family may coordinate and control the balance of life and death in the cells, therefore each protein likely only contribute partial effects.

Both NOXA and BIM are pro-apoptotic members of the BCL-2 protein family, and both can interact with MCL-1 and abolish the functions of MCL-1 [69]. Upregulation of NOXA can sensitize various cancer cell types to ABT-737 including melanoma [70–72]. TRAIL-induced MCL-1 inhibition leads to BIM-mediated apoptosis [73]. NOXA-MCL-1-BIM interplay is also important for apoptosis induced by drugs such as microtubule-targeting agents [74]. Our results also confirm the important roles of both proteins in antagonizing MCL-1 in melanoma, and that indeed NOXA-MCL-1-BIM axis can play a crucial role in the combination-induced killing of melanoma cells including the MICs.

The exciting CRISPR/Cas9 technology has advanced the gene-editing methodology tremendously, because it makes it possible to easily and inexpensively edit genetic information in virtually any organism, including human cells. This method also is more superior than shRNAs since you can knockout the gene of interest [55, 75]. We successfully used the CRISPR/Cas 9 technique to knock out BIM in our mechanistic study. Many recent studies suggest that off-target effects are rare for the CRISPR/Cas9 gene editing system [75–78]. In addition, we used two different gRNAs to delete BIM, and analyzed multiple BIM-null clones. Thus, it is very unlikely that the results obtained using these BIM-null lines are due to off-target effects.

A potentially significant anecdotal observation is that we have been unable to establish stable knockdown lines with a significant reduction in MCL-1 expression levels. Increasing the titer of the viral particles used significantly increases the level of killing during infection compared to shRNA vectors targeting other genes. We have also attempted to make MCL-1-null lines using CRISPR/Cas9 in three different melanoma cell lines using three different gRNAs against MCL-1. However, we could not find any clones lacking MCL-1 expression. Although indirect, this data further supports the idea that MCL-1 is essential for the survival of melanoma cells and is therefore an attractive therapeutic target for melanoma.

Designing a potent MCL-1 inhibitor is a huge challenge as directly inhibiting this target requires the disruption of high-affinity protein–protein interactions [79, 80]. SC-2001 is a derivative of Obatoclax, but both are small molecule BCL-2-, BCL-XL-, BCL-W-, and MCL-1 inhibitors which act on the intrinsic pathway [81]. Both compounds have been shown to inhibit MCL-1 by interrupting its interactions with other BCL-2 pro-apoptotic members [33, 82, 83]. Based on previous work and the work shown here, both compounds also act by increasing NOXA expression to antagonize MCL-1 [81, 82]. Obatoclax has been in clinical trials, but there are reports of some off-target effects [81]. Even though SC-2001 is reported to have better growth inhibition and death-inducing potential than Obatoclax in hepatocellular carcinoma cell lines [33], the study here can not completely rule out the possible contribution of off-target effects in our experiments. Recently, A-1210477 has been described as the most potent MCL-1 inhibitor available to date inducing clear on-target cellular activity. A-1210477 selectively disrupts MCL-1–NOXA and MCL-1–BIM complexes in living cells [80]. It was also demonstrated that A-121047 can synergize with the BCL-2 inhibitor ABT-263 in killing lung cancer, esophageal cancer, and multiple myeloma cell lines [80, 84]. It will be very interesting to study the clinical relevance and efficacy of this compound on killing melanoma cells and MICs.

Taken together, this work is of interest to both clinicians and researchers due to its following novelties: 1) Targeting MCL-1 and other BCL-2 family members is a promising approach to kill the bulk of melanoma cells, and most importantly, also the MICs. 2) This study also tested the effectiveness in melanoma cells from patients relapsed from current treatments, and in melanoma with wild type BRAF status which have fewer treatment options currently. 3) This study utilized the cutting-edge genomic-editing tool of CRISPR/Cas9 system to address the role of BIM in these treatments.

In summary, the data presented here suggests that MCL-1 is a promising target for treating melanomas and that SC-2001 is effective at killing non-melanoma-initiating cells. However, the combination treatment with both MCL-1 and BCL-2 inhibitors is capable of eliminating MICs which is needed to hinder relapse potential and block tumor regeneration. These treatments may be effective for melanoma regardless of the mutation status of BRAF or NRAS and may help overcome melanoma's resistance to current treatments. These results underline the need for developing potent and selective MCL-1 inhibitors that can be used in clinical trials.

## MATERIALS AND METHODS

### Reagents

SC2001 was synthesized as described previously in Chen et al.,[33]. ABT-737 was purchased from Selleck Chemicals.

### Cell lines, patient samples and culture conditions in monolayer and sphere cultures

Human melanoma metastatic cell lines A375, 1205Lu, SK-MEL-28, 451Lu, and HT-144 were obtained from ATCC (Manassas, VA) and WM852c was kindly provided by Dr. Meenhard Herlyn. NRAS mutated melanoma lines (SK-MEL-2, Hs852T) and patient-derived melanoma cell lines were provided by the Melanoma bank at University of Colorado Cancer center. Primary melanocytes were obtained from Life Technologies (Carlsbad, CA), and immortalized melanocytes PIG1 were kindly provided by Dr. Le Poole [85].

Cells were maintained in RPMI 1640 media (Invitrogen, Grand Island, NY) with 10% fetal bovine serum (Gemini Bio-Products, Inc., West Sacramento, CA). Melanocytes were maintained in Medium 254 with Human Melanocyte Growth Supplement-2 (Life Technologies, Carlsbad, CA). To mimic melanoma culture conditions, 10% FBS was added for drug assays.

All sphere assays were performed with poly-hema (Sigma, St. Louis, MO) coated plates or dishes [86], in stem cell media as described previously [27, 87, 88]. Specifically, the media contained DME/F12 (Hyclone) supplemented with B27 (Invitrogen), 20 ng/mL EGF and 20 ng/mL bFGF (BD Biosciences), and 4 μg/mL heparin (Sigma). Cells were seeded at the density of 1-5 viable cell/μl for melanoma cell lines and 10-20 viable cell/μl for the patient derived samples for all sphere assays.

Patient samples were only cultured in vitro for a short term (within 6 month of receipt) and some of the them were only maintained in a PDX model as described in [43, 89], so that they retain original tumor characteristics such as expression and mutation profiles. Xenografted tumors of F2-F4 generations were harvested and frozen as single-cell suspensions before being used in this study, and were prepared according to the method described in [89]. These patient samples were derived from melanoma biopsy samples of patients relapsed from various treatments. These melanoma cultures were validated by the bank with Melanoma Triple Cocktail staining. Melanoma Triple Cocktail (Ventana Medical Systems, Inc, Tucson, AZ) is an antibody cocktail of anti-Melanosome (HMB45), anti-MART-1/melan A (A103), and anti-Tyrosinase (T311) mouse monoclonal antibodies. The patient samples either harbored BRAF mutation (MB2309 and MB1823), NRAS mutation (MB1920 and PS4) or were triple-WT (wild type for BRAF, NRAS and NF1) (MB2141, and MB1374). The samples were collected after patients relapsed from the following treatments: Immunotherapy (MB1823) or BRAF-MEK inhibitors (MB2309) or multiple drugs/radiation/surgery (MB2141, MB1374).

### Measurement of cell proliferation, cytotoxicity, apoptosis and ALDH activity

The Cell Titer 96™ Aqueous One solution cell proliferation assay (ATP assay; Promega Corp., Madison, WI) was used to quantify cell viability, as instructed by the manufacturer. The cytotoxicity assay was conducted using the luminescent cyto-tox glo kit (Promega Corp., Madison, WI) in a 96 well format, as instructed by the manufacturer. Annexin V-FITC Apoptosis Detection Kit (BD Biosciences, San Jose, CA) was used to quantify apoptosis according to the manufacturer's protocol. Cells were analyzed by flow cytometry using a Beckman Coulter FC500 with CXP software (Hialeah, FL) in the University of Colorado Cancer Center Flow Cytometry Core. The Aldefluor kit (Stem Cell Technologies, Vancouver, Canada) was used to detect the ALDH activity according to the manufacturer's instructions. The Aldefluor staining was detected using the FITC channel and analyzed at the University of Colorado Cancer Center Flow Cytometry Core. At least three repeats were done for each cell line. The data was normalized as the relative fold in order to visualize the change of ALDH positive cells compared to the DMSO control, with the percentage of ALDH^high^ cells in DMSO condition set as “1”.

### Creation of short hairpin RNA transduced cell lines

Short hairpin RNA (shRNA) expressing cell lines against various BCL2 family members were created as described previously [23]. Knockdown of genes of interest was measured by immunoblotting of cell lysates.

### Creation of CRISPR mediated cell lines

BCL-2 family member BIM was knocked out by CRISPR /Cas9 technology. The protocol was followed from [54]. Briefly, the cells were first subjected to Cas-9 lentiviral transduction and then selected for Blasticydin resistance for 5 days. The Blasticydin-resistance Cas-9 transduced cell lines were then subjected to BIM gRNA lentiviral transduction. Functional Genomics Core at UC Boulder provided CRISPR/Cas9 related vectors, which were provided by Dr. Feng Zhang lab (The Broad Institute and the McGovern Institute of Brain Research at the Massachusetts Institute of Technology) [56]. Two different gRNA sequences of the lenti-guide puro-vectors are GCCCAAGAGTTGCGGCGTAT and CAACCACTATCTCAGTGCAA. After transduction, cells were selected with puromycin so that only cells transduced with a stable construct are preserved. The cells were then seeded in 96-well plate at the density of 1 cell/well using MoFlo XDP100 Cell sorter by the University of Colorado Cancer Center Flow Cytometry Core. The single cells were maintained for clonal expansion and each of the clones were expanded and tested to select for the complete knock-out, and screened and verified by immunoblotting of cell lysates.

### Immunoblot

Cells, both floating and adherent, were harvested with 1x Laemmli Sample Buffer (Bio-Rad, Hercules, CA). Samples were used in the standard western blot analysis protocol as described previously [90]. The following antibodies were used at suggested dilutions from the manufacturers: PARP1 (PARP), and TUBB2A (α/β Tubulin) (Cell Signaling Technology, Danvers, MA); PMAIP1 (NOXA, EMD Biosciences, Inc. San Diego, CA); MCL1 (BD Biosciences, San Jose, CA); BIM (Millipore, Billerica, MA), β-actin (Sigma Aldrich, St. Louis, MO) and HRP-conjugated goat anti-mouse and anti-rabbit antibodies (Jackson Immuno-Research, West Grove, PA). Immunoblots were typically performed 2-3 times for each cell line, and representative examples are shown. Immunoblot data was quantified using Image Studio Ver. 2.0 (LI-COR, Lincoln, NE).

### Primary and secondary sphere forming assays

#### Primary sphere assay

Cells were plated at a density of 1-20 viable cell/μl. Fresh media was added every 2-3 days. The spheres were allowed to grow up to reasonable size until Day 5 after seeding and then treated with indicated drugs on day 5. After 48 hrs, the numbers of spheres were counted and images captured using Nikon Eclipse TS100 scope fitted with Nikon DS-Fi2 camera.

#### Secondary sphere assay

Primary spheres, formed as mentioned above for indicated drug treatments, were dissociated into single cells and replated as described in [39, 41]. The procedures were the same as for the primary sphere assay, except that no drugs were added during the secondary sphere assays.

At least three repeats of both the primary and secondary sphere assays were done for each cell line. The data were normalized as the relative spheres in percentage compared to the vehicle (DMSO) control, and the number of spheres in the DMSO was set at “100”. The ethidium bromide/acridine orange stainingassay, as described previously [91, 92], was used to estimate live, dead, or apoptotic cells of the secondary spheres dissociated with PBS-EDTA [37].

### *In vivo* mouse xenograft studies

#### Conventional mouse xenograft study

Female NCRNU nude mice, aged 5 weeks, were used for the study. All animal experiments are approved by the Institutional Animal Care and Use Committee (IACUC) of the University of Colorado Denver (protocol number 88512(11)1E). Each mouse was subcutaneously injected on each flank with 1 million 1205Lu cells in a 100 μl volume consisting of 50% BD Matrigel Matrix (BD Biosciences) prepared according to the manufacturer's protocol. Drug treatments began after tumors reached approximately 100 mm^3^. Mice were randomly divided into two treatment groups consisting of at least 10 tumors each group: 1) vehicle only, 2) SC-2001 only. SC-2001 was administered (PO) at 10 mg/kg every alternate day, for 21 days. Mice were weighed daily and tumor volume was measured every alternate day with digital calipers.

#### MIC-mediated tumor xenograft

In this experiment, we employed a modified xenograft method with a low cell number implantation to test the differences in tumor initiating ability between the control and treatment groups. Melanoma sample MB1860 was used in this experiment. These cells were only maintained in the PDX model prior to this. The cells in the single-cell suspension were cultured in sphere condition, and treated with the indicated concentration of DMSO, single, or combined drug for 48hrs. Spheres were then dissociated into the single cell suspension and 50,000 viable cells were injected in each flank of nude mice and tumors were allowed to grow. Mice were weighed and tumor growth was measured as a readout for the impact of the single/combination treatment on tumor-initiation ability. Tumors were examined every alternate day. Tumor incidence was determined when the tumor was palpable and about 75 mm^3^ in size. Mice were sacrificed at the end of the experiment and tumors were collected and dissociated into single cell suspension to perform sphere assays. This modified xenograft method makes testing cancer initiation more feasible than the standard series dilution method. Similar approaches have been used previously in assessing tumor initiating ability in other cancer stem cell studies [49–52].

### Statistical analysis

All the graphs and statistical analyses for the ATP assay, IC50, sphere-forming assays and ALDH assay were created and conducted with GraphPad Prism 6 software. Specifically, One-Way Analysis of Variance (ANOVA) was used to evaluate if there were any statistically significant differences among all the conditions within each experiment. Tukey post-hoc test were then performed to determine which comparisons among the conditions was statistic significantly different. The analyses with P-value of 0.05 and below were considered significant. Survival curve is plotted as the percentage of tumor free incidence on indicated days and we used Log rank (Mantel-Cox) test for tumor incidence with Graphpad Prism 6 software. Statistical Analysis for tumor growth data was conducted using a mixed model followed by simple effect test for pairwise comparisons of mean fold change in tumor volume between treatment groups using SPSS software (IBM, SPSS Statistics). A P-value of 0.05 and less was considered significant. Data for tumor incidence was analyzed using ELDA software (WEHI bioinformatic resources) and significance was determined by chi-square analysis as described in [93, 94]. P-values of 0.05 and less was considered significant.

## SUPPLEMENTARY MATERIALS FIGURES AND TABLES



## Abbreviations

MIC: Melanoma Initiating Cells
CSC: Cancer Stem Cells
MCL-1: Myeloid cell leukemia sequence 1
BCL-2: B-cell CLL/lymphoma 2
BIM: BCL2-like 11 (apoptosis facilitator)
PARP: Poly ADP-ribose polymerase 1
KD: Knock Down
