# Supplementary material for: Use of a MCL-1 inhibitor alone to de-bulk melanoma and in combination to kill melanoma initiating cells
Source: Oncotarget. 2016 Apr 12;8(29):46801–17. doi: 10.18632/oncotarget.8695 (PMC5564524; doi:10.18632/oncotarget.8695)
Supplement: Supplementary file 1 [file oncotarget-08-46801-s001.pdf]

SUPPLEMENTARY FIGURES AND TABLES

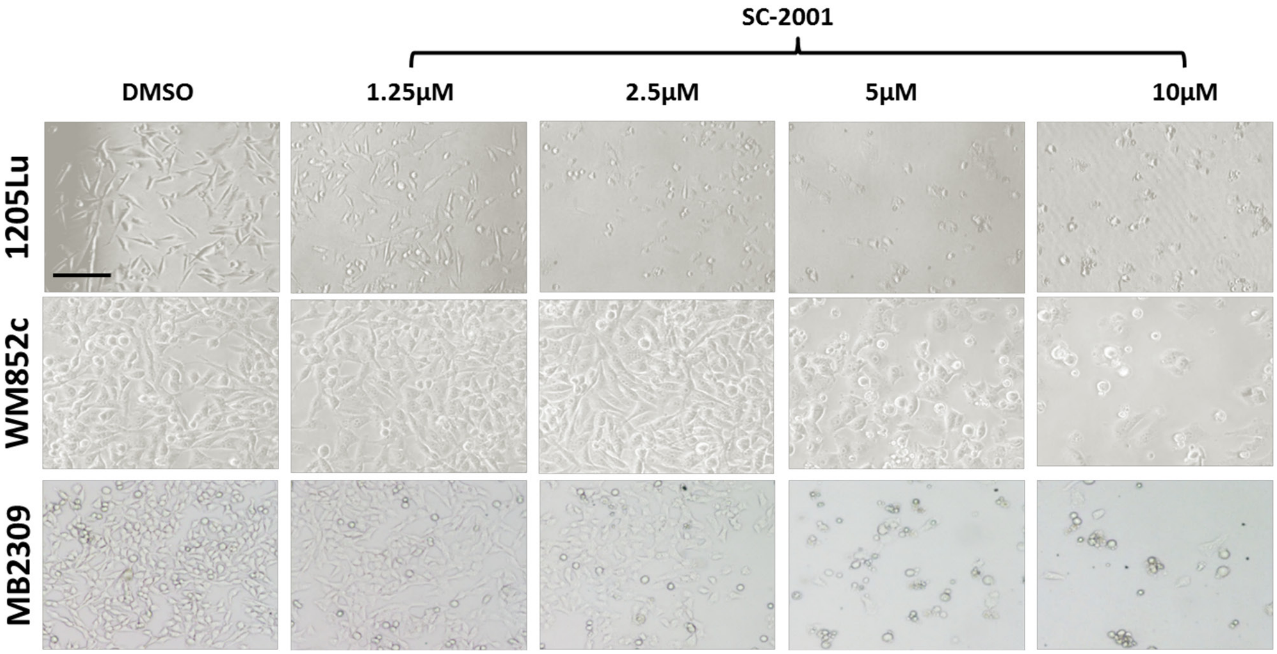

Supplementary Figure S1: Bright-field images of melanoma cells treated with indicated dose of SC-2001 for 48 hrs. Scale bar = 100µm.

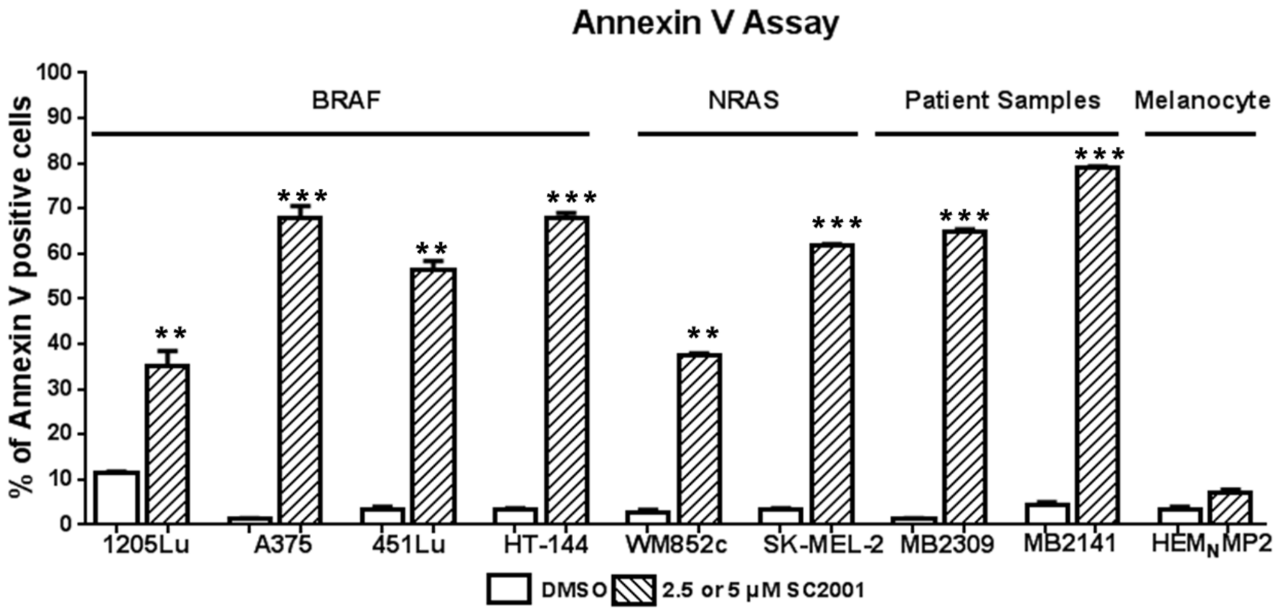

Supplementary Figure S2: The Annexin V assay shows massive apoptosis in melanoma cells induced by 48 hrs treatment of SC-2001. SC-2001 treatment significantly induced apoptosis compared to DMSO, (**P < 0.01; ***P < 0.001) in all melanoma cells tested (irrespective of the mutation status).

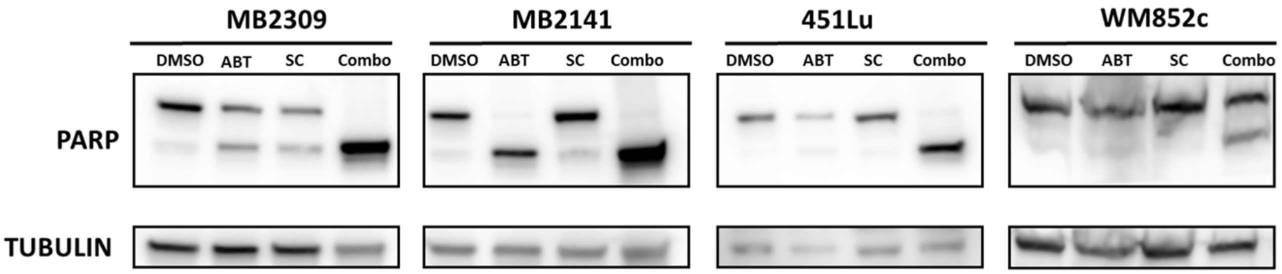

Supplementary Figure S3: Immunoblot of full length and cleaved PARP for sphere cell lysates treated with DMSO, ABT-737 (3.3µM), SC-2001 (2.5µM) or Combination for 48 hrs.

Supplementary Table S1: IC50 values for SC-2001

	IC50 (µM)
**A375**	3.52
**1205Lu**	4.61
**HT144**	2.14
**WM852c**	4.62
**MB2309**	4.12

Supplementary Table S2: Significance chart for Primary Sphere Assay

	DMSO vs. Combo	ABT-737 vs. Combo	SC-2001 vs. Combo
**A375**	***	***	**
**451Lu**	**	**	*
**SKMEL-28**	***	***	*
**HT144**	***	***	***
**1205Lu**	***	***	***
**WM852c**	***	***	**
**MB2141**	***	**	**
**MB2309**	**	**	***
**MB1823**	ns	ns	ns
**MB1374**	***	*	**
**MB1860**	***	***	**
**MB1468**	***	***	**
**MB1920**	***	***	***
**PS4**	***	***	***

***indicates P<0.001 or less; **indicates P<0.01; *indicates P<0.05

Supplementary Table S3: Significance chart for ALDH assay

	DMSO vs. Combo	ABT-737 vs. Combo
**A375**	***	*
**451Lu**	**	ns
**SKMEL-28**	***	**
**HT144**	*	*
**1205Lu**	**	*
**WM852c**	*	**
**MB2141**	***	***
**MB2309**	***	***

***indicates P<0.001 or less; **indicates P<0.01; *indicates P<0.05
Unfortunately, we did not have enough material to do additional replicates for last four samples of Figure 4C, so we could not statistically analyze the data

Supplementary Table S4: Significance chart for Secondary Sphere Assay

	DMSO vs. Combo	ABT-737 vs. Combo	SC2001 vs. Combo
**A375**	***	**	*
**451Lu**	***	***	***
**SKMEL-28**	***	***	*
**HT144**	*	*	*
**1205Lu**	***	***	***
**WM852c**	***	***	***
**MB2141**	***	***	***
**MB2309**	***	***	***
**MB1374**	ns	ns	ns

***indicates P<0.001 or less; **indicates P<0.01; *indicates P<0.05
